# Comparative Analysis of Proliferation and Differentiation Potentials of Stem Cells from Inflamed Pulp of Deciduous Teeth and Stem Cells from Exfoliated Deciduous Teeth

**DOI:** 10.1155/2014/930907

**Published:** 2014-06-22

**Authors:** Shi Yu, Shu Diao, Jinsong Wang, Gang Ding, Dongmei Yang, Zhipeng Fan

**Affiliations:** ^1^Laboratory of Molecular Signaling and Stem Cells Therapy, Beijing Key Laboratory of Tooth Regeneration and Function Reconstruction, Capital Medical University School of Stomatology, No. 4 Tiantanxili, Dongcheng District, Beijing 100050, China; ^2^Department of Pediatric Dentistry, Capital Medical University School of Stomatology, No. 4 Tiantanxili, Dongcheng District, Beijing 100050, China; ^3^Department of Biochemistry and Molecular Biology, Capital Medical University School of Basic Medical Sciences, Beijing 100069, China; ^4^Department of Stomatology, Yidu Central Hospital, Weifang Medical University, No. 4138 Linglong Mountain South Road, Qinzhou 262500, China

## Abstract

Stem cells isolated from exfoliated deciduous teeth (SHEDs) are highly capable of proliferation and differentiation, and they represent good cell sources for mesenchymal stem cell- (MSC-) mediated dental tissue regeneration, but the supply of SHEDs is limited. A previous study found that stem cells could be isolated from inflamed tissues, but it is unknown whether primary dental pulp diagnosed with irreversible pulpitis might contain stem cells with appropriate tissue regeneration capacity. In this study, we aimed to isolate stem cells from both inflamed pulps of deciduous teeth (SCIDs) and SHEDs from Chinese children and to compare their proliferation and differentiation potentials. Our results showed that SCIDs were positive for cell surface markers, including CD105, CD90, and CD146, and they had high proliferation ability and osteogenic, adipogenic, and chondrogenic differentiation potentials. There was no significant difference in proliferation and differentiation potentials between SCIDs and SHEDs. The mRNA of inflammatory factors, including IL-1*β*, IL-6, and TNF-*α*, was expressed at similar levels in SCIDs and SHEDs, but SCIDs secreted more TNF-*α* protein. In conclusion, our *in vitro* results showed that SCIDs have proliferation and differentiation potentials similar to those of SHEDs. Thus, SCIDs represent a new potentially applicable source for MSC mediated tissue regeneration.

## 1. Introduction

Emerging tissue engineering and stem cell-based therapies hold promise for great advances in regenerative medicine. Mesenchymal stem cells (MSCs) are considered a good cell source for tissue regeneration. MSC populations have been isolated from dental tissues, including the dental pulp, periodontal ligament, and dental follicle [1–3]. These cells are multipotent, show osteo-/dentinogenic differentiation, and are capable of self-renewal. Recently, MSCs have been identified in inflamed dental pulp, inflamed periodontal ligament, and inflamed periapical tissues [4–9]. Studies have shown that MSCs isolated from inflamed dental tissues retained their regeneration potential, but they exhibited a marked reduction in differentiation potential, particularly for mineralized tissue [4, 7]. Alongi et al. reported that inflamed pulp tissues contained a population of MSCs with diminished stem cell properties, including reduced osteo-/dentinogenic differentiation [4]. Similarly, Park et al. showed that inflamed human periodontal ligament stem cells possessed significantly reduced potential for forming cementum-like tissues, compared to stem cells from healthy periodontal tissue [7]. Compared to MSCs from noninflamed dental pulp and dental follicles, MSCs from periapical lesions showed lower clonogenicity and self-renewal rates [8]. However, other researchers have reported different findings [5, 6]. Wang et al. found that MSCs derived from tissues with irreversible pulpitis demonstrated low colony formation capacity and a slightly low cell proliferation rate, but their STRO-1 expression, their* ex vivo* osteogenic induction, and their dentin sialophosphoprotein expression were similar to those of STRO-1-enriched pulp cells [5]. Pereira et al. also isolated stem cells from dental pulp (DPSCs) and found that DPSCs derived from inflamed and normal tissues were similar in morphology, proliferation rates, and differentiation potentials. Thus, they demonstrated that the inflammatory process did not affect the stem cell properties assessed [6].

Stem cells from human exfoliated deciduous teeth (SHEDs) are a population of highly proliferative, clonogenic cells capable of differentiating into a variety of cell types, including neural cells, adipocytes, and odontoblasts [10–16]. The proliferation rate of SHEDs was significantly higher than that of DPSCs and bone marrow-derived mesenchymal stem cells (BMMSCs) [10–12]. Studies showed that SHEDs were capable of generating robust amounts of bone and pulp/dentin complexes* in vivo*, and they could alleviate Parkinson's disease [13–16]. However, due to the physiological absorption of roots in deciduous teeth, only a small amount of dental pulp remains in exfoliated deciduous teeth; thus, the supply of SHEDs is limited.

In clinical settings, a diagnosis of irreversible pulpitis is treated by removing the entire pulp tissue from primary teeth with a pulpectomy. In primary teeth, a large portion of pulp may be viable tissue that contains stem cells. Recent studies have reported that viable stem cells are present in inflamed pulp deciduous tissue. However, those cells had highly dysfunctional MSC characteristics, stemness, and immunomodulatory properties [17]. Another study elucidated that* in vitro* characteristics of MSCs, including growth, proliferation, and viability, were associated with* in vivo* functions of MSCs that are important for therapeutic use [18]. In the present study, we isolated stem cells from inflamed pulp of deciduous teeth (SCIDs) from Chinese children and then examined proliferation, differentiation potentials, and the expression of inflammatory factors. We compared these characteristics to those of SHEDs to investigate the regenerative potential of SCIDs.

## 2. Materials and Methods

### 2.1. Sample Collection and Cell Culture

Pulp tissues were obtained from primary teeth of patients (3–10 years of age) under approved guidelines set by Beijing Stomatological Hospital, Capital Medical University. All parents provided informed consent.

Exfoliated deciduous teeth were collected from 5 patients; all teeth were free of carious lesions. The pulps were separated from remnant crowns. Inflamed pulp of deciduous teeth was obtained by pulpectomy from 6 patients diagnosed with irreversible pulpitis. A portion of each inflamed pulp was fixed with 4% paraformaldehyde in PBS (pH 7.2) and stained with hematoxylin and eosin (HE) for pathological diagnosis.

All pulp samples were washed and digested in a solution of 3 mg/mL collagenase type I and 4 mg/mL dispase for 30–60 min at 37°C. Single cell suspensions were isolated and cultured as previously described [1–3]. Cells were grown in a humidified 5% CO_2_ incubator at 37°C in alpha modified Eagle's medium (MEM, Invitrogen, California, USA) supplemented with 15% fetal bovine serum (FBS; Invitrogen), 2 mmol/L glutamine, 100 U/mL penicillin, and 100 *μ*g/mL streptomycin (Invitrogen). The culture medium was changed every 3 days. SCIDs and SHEDs were used at passages 3–5 in subsequent experiments.

### 2.2. Characterization of SCIDs and SHEDs

SCIDs and SHEDs were labeled with fluorescein isothiocyanate-conjugated or phycoerythrin-conjugated antibodies and analyzed with flow cytometry. Cells from different individuals were mixed in equal proportions, and SCIDs and SHEDs were cultured separately. SCIDs and SHEDs were harvested with 0.25% trypsin, and cell aliquots (1.0 × 10^6^ cells) were incubated for 1 h at room temperature with monoclonal antibodies specific for CD90, CD105, or CD146 (BD Biosciences, USA) [1–3, 10, 17, 19]. Expression profiles were analyzed by flow cytometry (Calibur; BD Biosciences).

### 2.3. Cell Growth Curves and Population Doubling Time

MSCs were seeded at a density of 5.0 × 10^4^ cells/well into 6-well plates. Cells were counted at 3, 5, and 7 days after seeding. The results shown represent the mean values (±s.e.m.) of three separate experiments. To assess the population doubling time, cells were counted at 70–80% confluency. Population doubling time was calculated with the formula dt = *t* × ln2/ln(Ct/C0), where dt is the doubling time, *t* is the time between cell counts, and C0 and Ct (in hours) are the initial cell count and the cell count after time *t*, respectively.

### 2.4. Cell Counting Assay

MSCs were seeded at a density of 1.0 × 10^3^ cells/well into 96-well plates. Cells were grown in 96-well plates for 24 h or 48 h after seeding. Then, a cell counting kit solution (Cell Counting kit-8, Dojindo, Japan) was added to each well of the plate, and absorbance was measured at 450 nm, according to the manufacturer's protocol (Dojindo, Japan).

### 2.5. Alkaline Phosphatase Activity and Alizarin Red Staining

MSCs were seeded at a density of 2.0 × 10^5^ cells/well into 6-well plates with routine medium. When cells reached 80–90% confluence, the medium was changed, and cells were grown in mineralization-inducing medium with the STEMPRO osteogenesis differentiation Kit (Invitrogen). After induction, cells were fixed with 4% paraformaldehyde and stained with a solution of 0.25% naphthol AS-BI phosphate and 0.75% Fast red FRV with an alkaline phosphatase (ALP) staining kit, according to the manufacturer's protocol (Sigma-Aldrich). Then, ALP activity was measured with an ALP activity kit, according to the manufacturer's protocol (Sigma-Aldrich), and normalized to the protein concentrations. To detect mineralization, cells remained in the inducing medium for 2 weeks, and then they were fixed with 70% ethanol and stained with 2% Alizarin red (Sigma-Aldrich). To quantitatively measure calcium, Alizarin Red was destained with 10% cetylpyridinium chloride in 10 mM sodium phosphate for 60 min at room temperature. The calcium concentration was determined by measuring absorbance at 562 nm on a multiplate reader and comparing to a standard calcium curve prepared in the same solution. The final calcium level in each group was normalized to the total protein concentration, measured in a duplicate plate [19].

### 2.6. Oil Red O Staining

Adipogenic differentiation was induced with the STEMPRO Adipogenesis differentiation Kit (Invitrogen). MSCs were grown in the adipose-inducing media for 3 weeks. Next, cells were fixed with 10% formalin for at least 1 h at room temperature. Then, cells were stained with the Oil Red O working solution for 10 min. The proportion of Oil Red O-positive cells was determined by counting cells under a light microscope [20].

### 2.7. Alcian Blue Staining

Chondrogenic differentiation was induced with the STEMPRO chondrogenesis differentiation kit (Invitrogen). MSCs were grown in chondrogenic medium for 3 weeks. Then, cells were rinsed once with Dulbecco's PBS and fixed with 4% formaldehyde solution for 30 min. After fixation, cells were rinsed with Dulbecco's PBS, and then stained with 1% Alcian blue solution (prepared in 0.1 N HCl) for 30 min. Next, the cells were rinsed three times with 0.1 N HCl, and distilled water was added to neutralize the acidity. Cells were visualized under a light microscope, and images were captured for analysis. Blue staining indicated synthesis of proteoglycans by chondrocytes [20].

### 2.8. Real-Time Reverse Transcription-PCR

For real-time reverse transcription (RT)-PCR, 2 *μ*g aliquots of RNA were synthesized with random hexamers and reverse transcriptase, according to the manufacturer's protocol (Invitrogen). Real-time PCR was performed with the SYBR Green PCR kit (Qiagen, Germany) and an Icycler iQ Multi-color, Real-time PCR Detection System. The gene-specific primer sequences are shown in Supplementary Table 1 (in Supplementary Material available online at http://dx.doi.org/10.1155/2014/930907).

### 2.9. Enzyme Linked Immunosorbent Assay (ELISA)

MSCs were seeded at a density of 2.0 × 10^5^ cells/well into 6-well plates. At 48 h after seeding, the cells were spun down in a centrifuge, and the supernatant was collected to measure the cytokines. Expression levels of the cytokines TNF-*α*, IL-1*β*, and IL-6 were analyzed with separate ELISA kits for human TNF-*α*, IL-1*β*, and IL-6 cytokines (Dakewe Biotech Co., Ltd), according to the manufacturer's protocol. Each sample, standard, and blank was assayed in triplicate. First, 100 *μ*L of diluted Cytokine standard was added to standard wells, 100 *μ*L of sample into sample wells, and 100 *μ*L of blank solution into the blank wells. Then, 50 *μ*L of diluted biotinylated antibody was added into all wells. The plate was covered with a plate cover and incubated according to instructions. Next, 100 *μ*L of diluted streptavidin-HRP was added to all wells. After washing, 100 *μ*L of TMB substrate solution was pipetted into all wells, including the blank wells, and incubated in the dark for 30 min at 37°C. The enzyme-substrate reaction was stopped by quickly pipetting 100 *μ*L of Stop solution into each well. The OD values were measured at 450 nm within 15 min, with a multiplate reader. The results were calculated by comparing to the standards and blanks.

### 2.10. Statistical Analysis

All statistical calculations were performed with SPSSv.13.0 (SPSS Inc., Chicago, IL, USA) statistical software. Statistical significance was determined with the Student *t*-test. A *P* value ≤ 0.05 was considered significant.

## 3. Results

### 3.1. SCIDs Formed Colonies and Expressed CD90, CD105, and CD146

**Figure 1:**
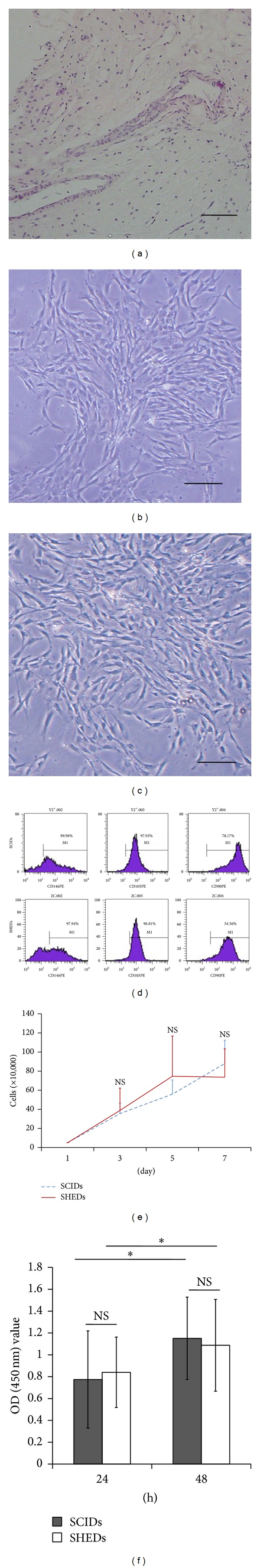
SCIDs formed colonies, expressed CD90, CD105, and CD146, and exhibited cell proliferation similar to SHEDs. (a) HE staining of inflamed primary pulp tissue shows lymphocyte cell infiltration and angiotelectasis. Scale bar: 100 *μ*m. (b) Cells derived from inflamed pulp formed colonies and showed typical fibroblast-like morphology. Scale bar: 10 *μ*m. (c) SHEDs formed colonies and showed typical fibroblast-like morphology. Scale bar: 10 *μ*m. (d) (left column) CD90, (middle column) CD105, and (right column) CD146 were expressed in both SCIDs (top row) and SHEDs (bottom row). (e) Cell growth curves showed that SCIDs and SHEDs had similar growth rates. Cell numbers were counted every two days for one week. The results represent the mean ± standard deviation from three independent experiments. The SCID cell numbers were not significantly different from SHED cell numbers. (f) Cell counting assay showed similar optical density (OD) values for SCIDs and SHEDs at 24 and 48 h. The results represent the mean ± standard deviation from three independent experiments. Student's *t*-test was performed to determine statistical significance. All error bars represent s.d. (*n* = 5). NS: no significant difference.

The SCIDs were derived from patients aged 4.7 ± 1.5 years and the SHEDs were derived from patients aged 8.4 ± 2.0 years. The primary pulps from irreversible pulpitis showed lymphocyte cell infiltration and angiotelectasis with HE staining (Figure 1(a)), which confirmed that these pulps were in an inflammatory state. Cells derived from the inflamed pulp could form colonies and showed typical fibroblast-like morphology (Figure 1(b)), similar to SHED cells (Figure 1(c)). SCIDs and SHEDs were both positive for CD90, CD105, and CD146 (Figure 1(d)).

### 3.2. Similar Cell Proliferation and Multidifferentiation Potentials for SCIDs and SHEDs

Cell proliferation was monitored over a period of 7 days after seeding. Cell growth curves showed that cell growth rates were similar for SCIDs and SHEDs at 1, 3, 5 and 7 days (Figure 1(e)). The cell counting assay showed no significant differences between SCIDs and SHEDs at 24 and 48 h (Figure 1(f)). Moreover, the average doubling times were not significantly different for SCIDs (38.3 ± 5.1 h) and SHEDs (38.5 ± 13.4 h). These results indicated that proliferation capacity was not significantly different between SCIDs and SHEDs.

**Figure 2:**
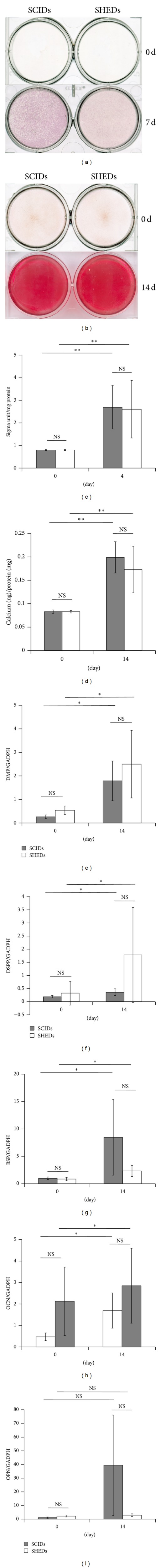
SCIDs and SHEDs showed similar osteo-/dentinogenic differentiation potentials. (a–d) Both SCIDs and SHEDs were cultured with osteo-/dentinogenic differentiation medium. (a) After 7 days, cells were stained with Fast Red Kit (Sigma-Aldrich). (c) ALP activities were similarly enhanced in SCIDs and SHEDs. (b) After 14 days, cells were stained with 2% Alizarin red. (d) Mineralization was similarly enhanced in SCIDs and SHEDs after osteo-/dentinogenic induction. (e, f) Real-time RT-PCR results indicated that dentinogenic makers, (e) DMP-1 and (f) DSPP, were similarly increased in SCIDs and SHEDs. GAPDH was used as an internal control. (g–i) Real-time RT-PCR showed that osteogenic markers, (g) BSP and (h) OCN, were similarly increased in SCIDs and SHEDs. (i) OPN expression was not significantly changed in SCIDs or SHEDs after culturing with osteogenic medium. Student's *t*-test was performed to determine statistical significance. All error bars represent s.d. (*n* = 4). ∗*P* < 0.05. ∗∗*P* < 0.01; NS: no significant difference.

Next, osteo-/dentinogenic differentiation potential was investigated by culturing SCIDs and SHEDs in osteogenic-inducing medium. ALP activity, an early marker for osteo-/dentinogenic differentiation, was similarly increased in SCIDs and SHEDs (Figures 2(a) and 2(c)). Two weeks after culturing the cells in osteogenic-inducing medium, alizarin red staining and quantitative calcium measurements revealed that mineralization was similarly enhanced in SCIDs and SHEDs after osteogenic induction (Figures 2(b) and 2(d)). Furthermore, real-time RT-PCR results showed that the expression levels of DSPP, DMP-1, BSP, and OCN were enhanced after 2 weeks of osteogenic induction (Figures 2(e)–2(h)); in contrast, OPN expression did not significantly change after 2 weeks of induction, for both SCIDs and SHEDs (Figure 2(i)). These real-time RT-PCR results on cell makers were consistent with our other findings in undifferentiated and differentiated SCIDs and SHEDs.

**Figure 3:**
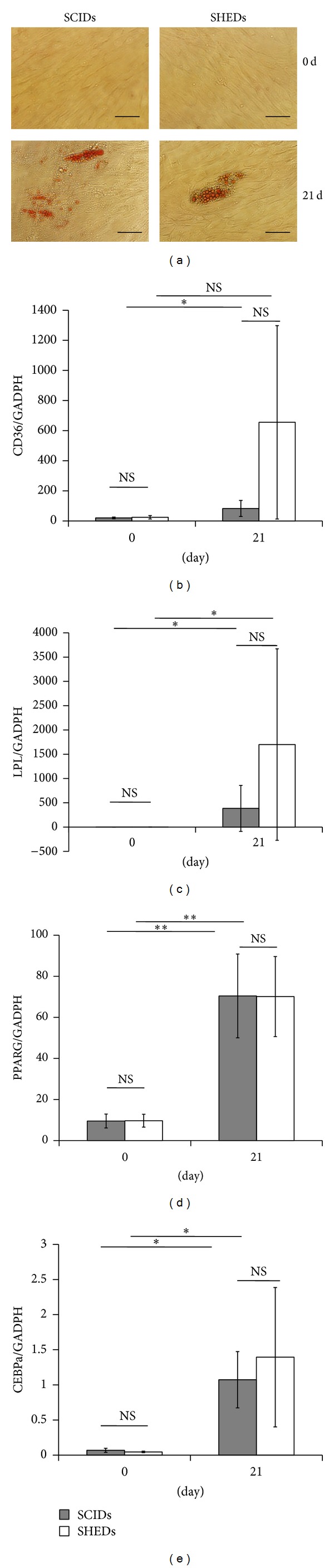
SCIDs and SHEDs showed similar adipogenic differentiation potentials. (a) SCIDs and SHEDs were cultured in adipogenesis differentiation medium for 3 weeks. Cells were stained with Oil Red O solution to visualize lipid deposits before (top, 0 d) and after (bottom, 21 d) adipogenic induction. Scale bar: 20 *μ*m. (b) CD36 expression was not significantly changed in SHEDs after adipogenic induction but significantly increased in SCIDs; however, the difference between SCIDs and SHEDs was not significant. (c–e) Real-time RT-PCR results showed similarly enhanced expression of (c) LPL, (d) PPARG, and (e) CEBPA in SCIDs and SHEDs after adipogenic induction. Student's *t*-test was performed to determine statistical significance. All error bars represent s.d. (*n* = 4). ∗*P* < 0.05. ∗∗*P* < 0.01. NS: no significant difference.

After induction with adipogenic medium for 3 weeks, oil red O staining revealed similar lipid deposits in SCIDs and SHEDs (Figure 3(a)). Real-time RT-PCR results showed that PPARG, CEBPA, and LPL expression could be induced after culturing in adipogenic medium for 3 weeks. SCIDs and SHEDs showed no significant difference in expression before (time = 0) and after 3 weeks of induction (Figures 3(c)–3(e)). However, after adipogenic induction, CD36 expression was increased in SCIDs, but not in SHEDs; nevertheless, the difference was not significant (Figure 3(b)).

**Figure 4:**
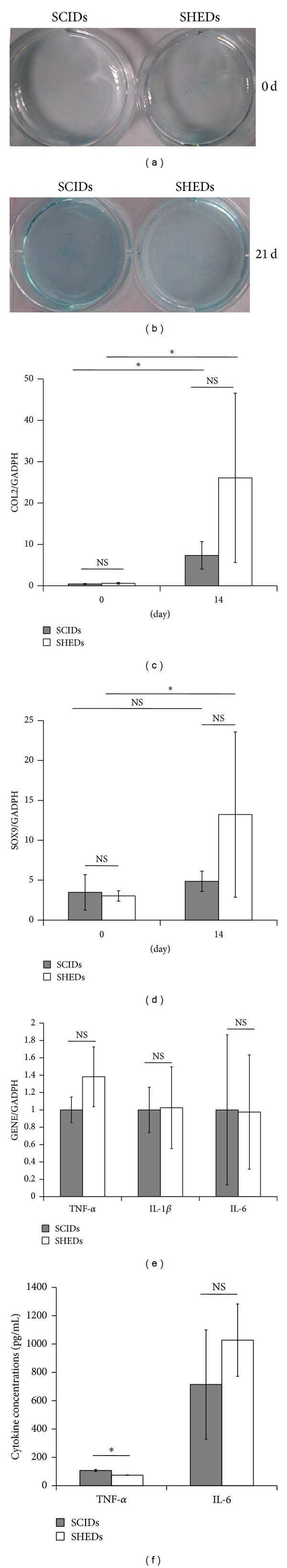
SCIDs and SHEDs showed similar chondrogenic differentiation potential, but SCIDs secreted more TNF-*α* protein than SHEDs. (a, b) Both SCIDs and SHEDs were cultured with chondrogenic differentiation medium for 3 weeks (21 d). Alcian blue staining revealed that proteoglycan production increased similarly in SCIDs and SHEDs. (c) Real-time RT-PCR results show significantly enhanced expression of COL2 in SCIDs and SHEDs after chondrogenic induction. (d) SOX9 mRNA detected with real-time RT-PCR in SHEDs and SCIDs before (0 d) and after (14 d) chondrogenic induction. Student's *t*-test was performed to determine statistical significance. (e) Real-time RT-PCR results show IL-1*β*, IL-6, and TNF-*α* expression. The expression levels were not significantly different between SCIDs and SHEDs. (f) IL-6 and TNF-*α* secretion levels were measured by ELISA. TNF-*α* secretion was significantly enhanced in SCIDs compared with SHEDs. IL-6 secretion was not different between SCIDs and SHEDs. All error bars represent s.d. (*n* = 4). ∗*P* < 0.05. ∗∗*P* < 0.01. NS: no significant difference.

After induction with chondrogenic medium for 3 weeks, Alcian blue staining revealed increased proteoglycan production in SCIDs and SHEDs (Figures 4(a) and 4(b)). Real-time RT-PCR showed that COL2 expression was induced after culturing in chondrogenic medium for 2 weeks, and the expression of these makers was not significantly different between SCIDs and SHEDs (Figure 4(c)). The expression of SOX9 was increased in SHEDs, but not in SCIDs, after chondrogenic induction. However, SOX9 expression levels were similar between SCIDs and SHEDs, both before and after differentiation (Figure 4(d)).

### 3.3. SCIDs Secreted More TNF-*α* Protein Than SHEDs

We monitored inflammatory cytokines, both at the mRNA level with real-time RT-PCR and at the protein level with ELISA. The results showed no significant differences between SCIDs and SHEDs in mRNA expression levels of IL-1*β*, IL-6, and TNF-*α* (Figure 4(e)). However, TNF-*α* protein secretion was significantly enhanced in SCIDs compared to SHEDs (Figure 4(f)); in contrast, SCIDs and SHEDs showed no significant difference in the secretion of IL-6 (Figure 4(f)). IL-1*β* secretion was undetectable in SCIDs and SHEDs in the ELISA assay (data not shown).

## 4. Discussion

Primary teeth that undergo pulpectomy are nearly free of root absorption, and the pulp tissues are nearly integrated. In contrast, exfoliated deciduous teeth typically have absorbed roots, and the remaining pulp tissues are limited. Thus, inflamed primary dental pulp may possess a large portion of viable pulp, but it remained unclear whether they contained stem cells with appropriate capacity for tissue regeneration.

In the present study, we isolated SCIDs and SHEDs and demonstrated that they both had the ability to form colonies, and they both could differentiate into osteo-/dentinoblasts, adipocytes, and chondrocytes. Previous studies have shown that MSCs* in vitro* showed growth, proliferation, and viability characteristics that accurately predicted MSC functions* in vivo* [18]. MSCs with good growth, proliferation, and viability were able to create vascularized, granulated tissues, and they supported long-term MSC engraftments. These discoveries strongly suggested that enhancing growth, proliferation, and viability in MSCs could enhance their potential for vascular and tissue regeneration. Our results showed that SCIDs and SHEDs possessed similar cell proliferation rates.

Considering the robust osteogenic ability of SHEDs and their successful application in dental and craniofacial regeneration, we focused on comparing osteo-/dentinogenic ability between SCIDs and SHEDs. We found that ALP activity and* in vitro* mineralization were not different between SCIDs and SHEDs.

Next, we compared the RNA expression levels of DSPP, DMP-1, BSP, OPN, and OCN in SCIDs and SHEDs. DSPP is secreted by odontoblasts throughout dentin formation, and it is cleaved into dentin sialoprotein and dentin phosphoprotein [21, 22]. It is considered a specific marker of odontogenic differentiation [22]. DMP1 is a noncollagenous protein expressed in mineralized tissues [23]. It is an early gene, expressed during the commitment of neural crest-derived cells into odontoblasts [24]. BSP is a major structural protein of the bone matrix, and OCN is the most abundant noncollagenous bone matrix protein. OPN is a major cell- and hydroxyapatite-binding protein synthesized by osteoblasts; it is involved in anchoring osteoclasts to the mineral of bone matrix, and it plays a critical role in bone remodeling [25, 26]. OPN is an essential factor in causing osteoporosis in postmenopausal women, because high OPN expression prevents osteogenesis; the counteraction of OPN may prove effective in activating osteoclasts [27]. Interestingly, our results showed that expression levels of DSPP, DMP-1, BSP, OPN, and OCN were not different between SCIDs and SHEDs.

In addition, compared to SHEDs, we found that SCIDs exhibited similar adipogenic differentiation potential, based on Oil red O staining and the expression of differentiation markers, including PPARG, CEBPA, LPL, and CD36. SCIDs and SHEDs also showed similar chondrogenic differentiation potential, based on Alcian blue staining and expression of differentiation markers, SOX9 and COL2. Taken together, our results indicated that SCIDs and SHEDs possessed similar cell proliferation and multidifferentiation potentials.

Previous studies have shown upregulation of various cytokines in inflamed pulp, including IL-1*β*, IL-2, IL-6, IL-8, IL-10, TNF-*α*, and INF-*γ* [28–30]. Root resorption in primary teeth and inflammatory propagation were both shown to be initiated and regulated by the secretion of stimulatory molecules, including cytokines and transcription factors. However, based on our real-time PCR results, we found that SCIDs and SHEDs expressed similar mRNA levels of IL-1*β*, IL-6, and TNF-*α*. ELISA results showed that SCIDs and SHEDs secreted similar amounts of IL-6 protein into the culture medium, but SCIDs secreted more TNF-*α* protein compared to SHEDs. TNF-*α* is involved in a wide spectrum of biological processes, including cell proliferation, differentiation, apoptosis, lipid metabolism, and coagulation. Several studies have reported that TNF-*α* promoted odontogenic/osteogenic differentiation in DPSCs [31–33]. TNF-*α* activated the NF-*κ*B pathway during osteogenic differentiation and increased mineralization; in addition, it upregulated the expression levels of bone morphogenetic protein 2, ALP, runt-related transcription factor 2, and collagen type I [33]. However, earlier reports had suggested that TNF-*α* was a negative regulator of osteoblast differentiation [34–36] and that long-term treatment with TNF-*α* could inhibit tooth mineralization. It is possible that TNF-*α* has different short-term and long-term effects on MSCs and/or different effects at different developmental stages of MSCs; thus, the effect of TNF-*α* on SCIDs may be difficult to predict. These issues will require further study for elucidation.

Recently, it was controversial whether MSCs isolated from inflamed dental tissues would retain the regeneration potential observed in MSCs from normal dental tissues [4–8]. Yazid et al. reported that MSCs derived from inflamed pulp deciduous tissues were highly dysfunctional in MSC characteristics, stemness, and immunomodulatory properties [17]. In contrast, our results showed that there were no significant differences between SCIDs and SHEDs in their* in vitro* proliferation and multidifferentiation potentials. In the Yazid study, patient ages were similar in groups with healthy and inflamed deciduous pulp tissues [17]. However, in general, patients diagnosed with irreversible pulpitis in primary teeth are typically younger than those with exfoliated deciduous teeth. Therefore, in the present study, the mean age of the SHED group was significantly older than that of the SCID group. Previous studies have shown that there is an age-related decline in cell functions [37–40]. Several reports have indicated that a reduction in proliferative ability was strongly correlated with increasing age. For example, DPSCs derived from younger individuals showed faster doubling times than those from senior individuals [39]. In addition, with increasing age, dental stem cells underwent a decline in their capacity to perform neurogenic differentiation but an increase in their capacity to perform osteogenic differentiation* in vitro* [40, 41]. Nevertheless, compared to DPSCs from young donors, aged DPSCs showed retarded pulp regeneration after autologous transplantation* in vivo* [42]. The aging microenvironment was demonstrated to have an inhibitory effect on adult stem cells [41–44]. Indeed, conditioned medium derived from MSCs showed a decline, with increasing age, in its abilities to enhance proliferation, differentiation, and migration [41, 42, 44]. However, there is a lack of information about age-related changes in stem cells from deciduous teeth, particularly when the aging period of interest is relatively short, compared to age-related changes in stem cells from permanent teeth.

In conclusion, our study verified that SCIDs showed high proliferative capacity and osteogenic, adipogenic, and chondrogenic differentiation potential. There were no significant differences between SCIDs and SHEDs in their proliferation and multidifferentiation potentials* in vitro*. Our results suggested that SCIDs might represent a new, viable source of cells for MSC-mediated tissue regeneration applications.

## Supplementary Material

GAPDH was used as an internal control. BSP, OPN, OCN, DSPP and DMP-1 were osteo-/dentinogenic-related makers. CD36, LPL, PPARG and CEBPA were adipogenic-related markers. COL2 and SOX9 were markers of chondrogenic differentiation. IL1B, IL6 and TNFA were inflammatory cytokines.

## Abbreviations

MSCs: Mesenchymal stem cells
SHEDs: Stem cells from human exfoliated deciduous teeth
SCIDs: Stem cells from human inflamed pulp of deciduous teeth
DPSCs: Dental pulp stem cells
BMMSCs: Bone marrow-derived mesenchymal stem cells
ALP: Alkaline phosphatase
GAPDH: Glyceraldehyde-3-phosphate dehydrogenase
BSP: Bone sialoprotein
OPN: Osteopontin
OCN: Osteocalcin
DSPP: Dentin sialophosphoprotein
DMP-1: Dentin matrix protein 1
CD36: CD36 molecule
CEBPA: CCAAT/enhancer binding protein (C/EBP), alpha
LPL: Lipoprotein lipase
PPARG: Peroxisome proliferator-activated receptor gamma
SOX9: Sex determining region Y-box 9
COL2: COL2A1 collagen, type II, alpha 1
IL-1*β*: Interleukin 1*β*
IL-6: Interleukin 6
TNF-*α*: Tumor necrosis factor *α*
CD90 (THY-1): Thy-1 cell surface antigen
CD105 (ENG): Endoglin
CD146 (NCAM): Melanoma cell adhesion molecule.
