# Supplementary material for: Comparative Analysis of Proliferation and Differentiation Potentials of Stem Cells from Inflamed Pulp of Deciduous Teeth and Stem Cells from Exfoliated Deciduous Teeth
Source: Biomed Res Int. 2014 Jun 22;2014:930907. doi: 10.1155/2014/930907 (PMC4090480; doi:10.1155/2014/930907)
Supplement: Supplementary file 1 — GAPDH was used as an internal control. BSP, OPN, OCN, DSPP and DMP-1 were osteo-/dentinogenic-related makers. CD36, LPL, PPARG and CEBPA were adipogenic-related markers. COL2 and SOX9 were markers of chondrogenic differentiation. IL1B, IL6 and TNFA were inflammatory cytokines. [file 930907.f1.docx]

| GENE SYMBOL | PRIMER SEQUENCES (5'--3') |
| --- | --- |
| GAPDH-F | CGAACCTCTCTGCTCCTCCTGTTCG |
| GAPDH-R | CATGGTGTCTGAGCGATGTGG |
| BSP-F | CAGGCCACGATATTATCTTTACA |
| BSP-R | CTCCTCTTCTTCCTCCTCCTC |
| OPN-F | ATGATGGCCGAGGTGATAGT |
| OPN-R | ACCATTCAACTCCTCGCTTT |
| OCN-F | AGCAAAGGTGCAGCCTTTGT |
| OCN-R | GCGCCTGGGTCTCTTCACT |
| DSPP-F | CGACATAGGTCACAATGAGGATGTCG |
| DSPP-R | TTGCTTCCAGCTACTTGAGGTC |
| DMP-1-F | CGTGGACAAAGAAGATAGCAACTCCACG |
| DMP-1-R | TTCCGGCTCTCTATCTCAATGTTT |
| CD36-F | CGATTAACATAAGTAAAGTTGCCATAATCG |
| CD36-R | CGCAGTGACTTTCCCAATAGGAC |
| LPL-F | CGGATTAACATTGGAGAAGCTATCCG |
| LPL-R | AGCTGGTCCACATCTCCAAGTC |
| PPARG-F | CGAGACCAACAGCTTCTCCTTCTCG |
| PPARG-R | TTTCAGAAATGCCTTGCAGTGG |
| CEBPA-F | CGGCTTATCCTAAATACTAGAGTTGCCG |
| CEBPA-R | GGACTTGGTGCGTCTAAGATGA |
| COL2-F | CATCCCACCCTCTCACAGTT |
| COL2-R | TCTGCCCAGTTCAGGTCTCT |
| SOX9-F | CCCTTCAACCTCCCACACTA |
| SOX9-R | TGGTGGTCGGTGTAGTCGTA |
| IL1B-F | CGAATGCTAAGAAACCCTCTGTCATTCG |
| IL1B-R | AATAAATAGGGAAGCGGTTGCTCA |
| IL6-F | CGCAACAACTCATCTCATTCTGCG |
| IL6-R | CATGCTACATTTGCCGAAGAGC |
| TNFA-F | CTACAGCGATGTCAGGGATCAAAGCTGTAG |
| TNFA-R | CACAGTGAAGTGCTGGCAACC |

**Supplementary** **table1. Primers sequences used in the real-time RT-PCR**
